# TRPA1 mediates the antinociceptive properties of the constituent of *Crocus sativus* L., safranal

**DOI:** 10.1111/jcmm.14099

**Published:** 2019-01-12

**Authors:** Simone Li Puma, Lorenzo Landini, Sergio J. Macedo, Viola Seravalli, Ilaria M. Marone, Elisabetta Coppi, Riccardo Patacchini, Pierangelo Geppetti, Serena Materazzi, Romina Nassini, Francesco De Logu

**Affiliations:** ^1^ Department of Health Sciences Section of Clinical Pharmacology and Oncology University of Florence Florence Italy; ^2^ Department of Pharmacology Federal University of Santa Catarina Florianópolis Brazil; ^3^ Department of Health Sciences Section of Paediatrics, Midwifery, Gynaecology and Nursing University of Florence Florence Italy; ^4^ Department of Neuroscience, Psychology, Drug Research and Child Health Section of Pharmacology and Toxicology University of Florence Florence Italy; ^5^ Department of Pharmacology Chiesi Farmaceutici SpA Parma Italy

**Keywords:** calcitonin gene‐related peptide, neurogenic inflammation, pain, safranal, transient receptor potential ankyrin 1

## Abstract

Safranal, contained in *Crocus sativus* L., exerts anti‐inflammatory and analgesic effects. However, the underlying mechanisms for such effects are poorly understood. We explored whether safranal targets the transient receptor potential ankyrin 1 (TRPA1) channel, which in nociceptors mediates pain signals. Safranal by binding to specific cysteine/lysine residues, stimulates TRPA1, but not the TRP vanilloid 1 and 4 channels (TRPV1 and TRPV4), evoking calcium responses and currents in human cells and rat and mouse dorsal root ganglion (DRG) neurons. Genetic deletion or pharmacological blockade of TRPA1 attenuated safranal‐evoked release of calcitonin gene‐related peptide (CGRP) from rat and mouse dorsal spinal cord, and acute nociception in mice. Safranal contracted rat urinary bladder isolated strips in a TRPA1‐dependent manner, behaving as a partial agonist. After exposure to safranal the ability of allyl isothiocyanate (TRPA1 agonist), but not that of capsaicin (TRPV1 agonist) or GSK1016790A (TRPV4 agonist), to evoke currents in DRG neurons, contraction of urinary bladder strips and CGRP release from spinal cord slices in rats, and acute nociception in mice underwent desensitization. As previously shown for other herbal extracts, including petasites or parthenolide, safranal might exert analgesic properties by partial agonism and selective desensitization of the TRPA1 channel.

## 1 INTRODUCTION

*Crocus sativus* L., known as saffron crocus, belongs to the family of *Iridaceas* 1 and is commonly used for flavouring and colouring food preparations. Saffron extracts contain three main bioactive constituents: the carotenoid crocin, responsible for its typical colour, the monoterpene aldehyde picrocrocin, and the volatile compound safranal, which accounts for its special flavour.^2^ Saffron has been reported to possess beneficial effects against depression, sexual dysfunction, premenstrual syndrome and weight loss.^1^, ^3^ Although clinical trials reported headache as one possible adverse effect of saffron,^4^ in Indian traditional medicine saffron has been used to treat headache.^5^ Preclinical studies focused on the pharmacological activity of saffron and its purified constituents, suggesting anti‐oxidant,^6^ anti‐inflammatory^7^ and antinociceptive^8^ properties of the golden spice. Antinociceptive effect of safranal has been demonstrated in models of inflammatory pain, such as formalin, acetic acid or carrageenan,^8^ and in models of neuropathic pain, including chronic constriction injury and nerve crush injury.^9^, ^10^ The analgesic action of safranal has been attributed to its ability to suppress glial activation and proinflammatory cytokines production in the central nervous system.^11^ However, the underlying mechanisms responsible for the analgesic action of saffron have not been yet elucidated.

Transient receptor potential (TRP) channels are pleiotropic excitatory ion channels present in a large variety of cells. Some TRPs are highly expressed by specific subpopulations of primary sensory nociceptive neurons, where they contribute to sensing noxious chemical, mechanical and thermal stimuli.^12^, ^13^, ^14^ In particular, the TRP ankyrin 1 (TRPA1) is coexpressed with the vanilloid type 1 (TRPV1, the capsaicin receptor), or the vanilloid type 4 (TRPV4) by a subpopulation of nociceptors, which contain and release the proinflammatory neuropeptide calcitonin gene‐related peptide (CGRP) and substance P (SP).^12^, ^13^ CGRP and SP released from peripheral endings of primary afferents mediate neurogenic inflammation^13^ and CGRP is now recognized to play an essential role in migraine pain.^13^, ^15^ TRPA1 is activated by a number of exogenous irritant and pungent agents, including allyl isothiocyanate (AITC), cinnamaldehyde^16^, ^17^ and formalin.^18^ Notably, reactive oxygen, nitrogen and carbon species gate TRPA1 by reacting with specific cysteine/lysine residues, thus mediating pain and neurogenic inflammation.^19^, ^20^, ^21^, ^22^, ^23^

Here, we evaluated whether the three main constituents of saffron affect the function of TRP channels expressed in nociceptors, and in particular that of TRPA1. We found that safranal and its precursor, picrocrocin, but not crocin, selectively activate TRPA1, but not TRPV1 or TRPV4, safranal being far more effective than picrocrocin. In addition, safranal behaves as a partial agonist on TRPA1 as compared to the full agonist, AITC. Finally, safranal induces selective desensitization of the TRPA1 channel, thus attenuating neuronal excitation that results in nociception and CGRP release. This unforeseen TRPA1‐desensitization mechanism could possibly explain the analgesic effects attributed to saffron.

## 2 MATERIALS AND METHODS

### 2.1 Animals

In vivo experiments and tissue collection were carried out according to the European Union (EU) directive guidelines (2010/63/EU) and Italian legislation (DLgs 26/2014) for animal care procedures. Studies were conducted under the University of Florence research permit #194/2015‐PR. Animals were housed in a temperature‐ and humidity‐controlled vivarium (12‐hour dark/light cycle, free access to food and water) and were allowed to acclimatize for at least 7 days prior to experimentation. Animal studies were reported in compliance with the ARRIVE guidelines.^24^, ^25^ A group size of six animals for behavioural experiments was determined by sample size estimation using G*Power (v3.1)^26^ to detect size effect in a post‐hoc test with type 1 and 2 error rates of 5 and 20% respectively. Allocation concealment was performed using a randomization procedure (http://www.randomizer.org/).

The following mouse strains were used: C57BL/6 mice (male, 20‐25 g, 5 weeks; Envigo, Milan, Italy); littermate TRPA1 wild‐type (*Trpa1* ^+/+^) and TRPA1‐deficient (*Trpa1* ^−/−^) mice (25‐30 g, 5‐8 weeks) generated by heterozygotes on a C57BL/6 background (B6.129P‐*Trpa1* ^tm1Kykw/J^; Jackson Laboratories, Bar Harbor, ME, USA); wild‐type (*Trpv4* ^+/+^) and TRPV4‐deficient (*Trpv4* ^−/−^) mice (25‐30 g, 5‐8 weeks), generated by heterozygotes on a C57BL/6 background^27^ and TRPV1‐deficient mice (*Trpv1* ^−/−^; B6.129X1‐*Trpv1* ^tm1Jul/J^) backcrossed with C57BL/6 mice (*Trpv1* ^+/+^) for at least 10 generations (Jackson Laboratories; 25‐30 g, 5‐8 weeks). Sprague‐Dawley rats (male, 75‐100 g, Envigo) were also used. Animals were killed with inhaled CO_2_ plus 10%‐50% O_2_.

### 2.2 Cell culture

Naïve untransfected HEK293 cells (American Type Culture Collection, Manassas, VA, USA; ATCC^®^ CRL‐1573™) were cultured according to the manufacturer's instructions. HEK293 cells were transiently transfected with the cDNAs (1 μg) codifying for wild‐type (wt‐hTRPA1) or mutant 3C/K‐Q human TRPA1 (C619S, C639S, C663S, K708Q; 3C/K‐Q hTRPA1‐HEK293)^23^ using the jetPRIME transfection reagent (Poliyplus‐transfection^®^ SA, Strasburg, France) following the manufacturer's protocol. HEK293 cells stably transfected with cDNA for human TRPA1 (hTRPA1‐HEK293), or with cDNA for human TRPV1 (hTRPV1‐HEK293), or with cDNA for human TRPV4 (hTRPV4‐HEK293) were cultured as previously described.^28^ Human foetal lung fibroblasts (IMR90; American Type Culture Collection; ATCC^®^ CCL‐186™), which express the native TRPA1 channel, were cultured as previously described.^29^ Cells were plated on glass‐coated (poly‐l‐lysine, 8.3 μmol/L) coverslips and cultured for 1‐2 days before being used for recordings.

### 2.3 Isolation of primary sensory neurons

Primary dorsal root ganglion (DRG) neurons were isolated from adult Sprague‐Dawley rats and *Trpa1* ^+/+^ and *Trpa1* ^−/−^ mice, and cultured as previously described.^30^ Briefly, DRG were bilaterally excised and transferred to Hank's Balanced Salt Solution containing trypsin (1 mg/mL) and collagenase type 1A or papain (both, 2 mg/mL), for rat or mouse ganglia respectively (35 minutes, 37°C). Ganglia were then transferred to warm Dulbecco's Modified Eagle Medium supplemented with 10% foetal bovine serum, 10% horse serum, 2 mmol/L L‐glutamine, 100 U/mL penicillin and 100 mg/mL streptomycin and dissociated into single cells by several passages through a series of syringe needles (23‐25G). Ganglia cells were centrifuged and suspended in medium with the addition of normal growth factor (100 ng/mL) and cytosine‐d‐arabinofuranoside free base (2.5 mmol/L). Neurons were then plated on glass coverslips coated with poly‐l‐lysine (8.3 μmol/L) and laminin (5 μmol/L).

### 2.4 Cellular recordings

Mobilization of intracellular calcium ([Ca^2+^]_i_) was measured in untrasfected or transfected HEK293 cells, in IMR90 and in DRG neurons. Cells on coated coverslips were loaded with 5 μmol/L Fura‐2 AM‐ester (Alexis Biochemicals, Lausen, Switzerland) added to the extracellular solution (37°C) containing the following (in mmol/L): 2 CaCl_2_, 5.4 KCl, 0.4 MgSO_4_, 135 NaCl, 10 D‐glucose, 10 HEPES and 0.1% bovine serum albumin at pH 7.4. After 40 minutes loading, coverslips with cells were washed and then transferred to a chamber on the stage of an Olympus IX81 microscope for recording. Cells were excited alternatively at 340 and 380 nm and recorded with a dynamic image analysis system (XCellence Imaging software; Olympus srl, Milan, Italy). Results were expressed as the percentage of increase in R_340/380_ over the baseline (% Change in R_340/380_), and each effect was normalized to the maximum effect induced by ionomycin (5 μmol/L).^30^ For whole‐cell patch‐clamp recordings, coverslips with cells were transferred to a recording chamber (1 mL volume), mounted on the platform of an inverted microscope (Olympus CKX41) and superfused at a flow rate of 2 mL/min with a standard extracellular solution at pH 7.4 (adjusted with NaOH) containing (in mmol/L): 10 HEPES, 10 D‐glucose, 147 NaCl, 4 KCl, 1 MgCl_2_, and 2 CaCl_2_. Borosilicate glass electrodes (Harvard Apparatus, Holliston, MA, USA) were pulled with a Sutter Instruments puller (model P‐87) to a final tip resistance of 4‐7 MΩ. Pipette solution used for HEK293 cells contained (in mmol/L): 134 K‐gluconate, 10 KCl, 11 EGTA, 10 HEPES (pH adjusted to 7.4 with KOH). When recordings were performed on rat DRG neurons, the extracellular solution contained 5 mmol/L CaCl_2,_ and pipette solution contained (in mmol/L): 120 CsCl, 3 Mg_2_ATP, 10 BAPTA, 10 HEPES‐Na (pH adjusted to 7.4 with CsOH). Data were acquired with an Axopatch 200B amplifier (Axon Instruments, CA, USA), stored and analysed with a pClamp 9.2 software (Axon Instruments). All the experiments were carried out at room temperature (20‐22°C). Currents were detected as inward currents activated on cell superfusion with the various stimuli in the voltage‐clamp mode (holding potential of −60 mV). Cell membrane capacitance was calculated in each cell throughout the experiment by integrating the capacitive currents elicited by a ±10 mV voltage pulse. Peak currents were normalized to cell membrane capacitance and expressed as mean of the current density (pA/pF) in averaged results. Signals were sampled at 1 kHz and low‐pass filtered at 10 kHz.

Cells and neurons were challenged with safranal (0.1‐300 μmol/L), picrocrocin (1‐300 μmol/L) and crocin (30‐200 μmol/L). Allyl‐isothiocyanate (AITC, 1‐10 μmol/L) and GSK1016790A (0.05‐0.1 μmol/L) were used to induce TRPA1 and a TRPV4 selective response respectively. Capsaicin (0.1‐1 μmol/L) was used to induce a TRPV1 selective response and to identify capsaicin‐sensitive neurons. Buffer solution containing dimethyl sulfoxide (DMSO, 1%) was used as vehicle. The activating peptide for human proteinase‐activated receptor 2 (hPAR2‐AP; 100 μmol/L) or KCl (40‐80 mmol/L) were used to elicit a TRP‐independent cellular response. Some experiments were performed in the presence of TRPA1, TRPV1 and TRPV4 selective antagonists, HC‐030031 (50 μmol/L), capsazepine (10 μmol/L) and HC‐067047 (30 μmol/L) respectively, or their vehicles (0.5% or 0.1% or 0.3% DMSO respectively).

### 2.5 Organ bath assay

Rat urinary bladder was excised from rat, and longitudinal strips were suspended at a resting tension of 1 g in 10‐mL organ bath bathed in aerated (95% O_2_ and 5% CO_2_) Krebs‐Henseleit solution maintained at 37°C containing (in mmol/L): 119 NaCl, 25 NaHCO_3_, 1.2 KH_2_PO_4_, 1.5 MgSO_4_, 2.5 CaCl_2_, 4.7 KCl and 11 D‐glucose. After 40 minutes of equilibration, tissues were challenged twice with carbachol (CCh, 1 μmol/L), with a 45‐minute washing out period between the two administrations. Motor activity was recorded on a force transducer isometrically (Harvard Apparatus, Ltd, Kent, UK). Tissues were challenged with safranal (10‐300 μmol/L), AITC (100 μmol/L), GSK1016790A (10 μmol/L) and capsaicin (0.3 μmol/L) or their vehicles. In some experiments, tissues were pre‐exposed to HC‐030031 (50 μmol/L), capsazepine (10 μmol/L), HC‐067047 (30 μmol/L) or a combination of NK1 and NK2 receptor antagonists, L‐733,060 and SR48968 respectively (both 1 μmol/L). Some preparations were desensitized by treatment with a high concentration of capsaicin (10 μmol/L for 20 minutes, twice) or were exposed to safranal (300 μmol/L for 20 minutes, twice) before the challenge with other stimuli. Responses were expressed as percentage (%) of the maximum contraction, induced by CCh (1 μmol/L).

### 2.6 CGRP‐like immunoreactivity assay

For CGRP‐like immunoreactivity (CGRP‐LI) outflow, 0.4‐mm slices of rat or mouse spinal cord were superfused with an aerated (95% O_2_ and 5% CO_2_) Krebs‐Henseleit solution modified with 0.1% bovine serum albumin plus the angiotensin‐converting enzyme inhibitor, captopril (1 μmol/L) and the neutral endopeptidase inhibitor, phosphoramidon (1 μmol/L) to minimize peptide degradation. Fractions (4 mL) of superfusate were collected at 10‐minute intervals before, during and after administration of the stimuli and then freeze‐dried, reconstituted with assay buffer, and analysed for CGRP‐LI using a commercial enzyme‐linked immunosorbent assay kit (Bertin Pharma, Montigny le Bretonneux, France). Detection limits of the assays were 5 pg/mL. Stimuli did not cross‐react with CGRP antiserum. Tissues were stimulated with safranal (10‐300 μmol/L) or its vehicle (1% DMSO). Some tissues were pre‐exposed to capsaicin (10 μmol/L, 30 minutes) or superfused with a calcium‐free buffer containing EDTA (1 mmol/L). Some preparations were pre‐exposed to TRPA1 antagonist, HC‐030031 (50 μmol/L), capsazepine (10 μmol/L) or HC‐067047 (30 μmol/L). Other tissues were pre‐exposed to safranal (100 μmol/L, 30 minutes) and then, after a prolonged washing (40 minutes), stimulated with AITC (50 μmol/L) capsaicin (0.1 μmol/L) or GSK1016790A (10 μmol/L). Results were expressed as femtomoles of peptide *per* gram of tissue.

### 2.7 Acute nociceptive response

The acute nociception was assessed in C57BL/6, *Trpa1* ^+/+^ and *Trpa1* ^−/−^, *Trpv1* ^+/+^ and *Trpv1* ^−/−^ *or Trpv4* ^+/+^ and *Trpv4* ^−/−^ mice, after intraplantar (i.pl.) injection (20 μL/paw) of safranal (0.2‐20 nmol), AITC (10 nmol), capsaicin (0.2 nmol), GSK1016790A (2 nmol) or their vehicle (7% and 0.5% DMSO). Immediately after injection, mice were placed individually in plexiglas chambers and the amount of time (seconds) spent licking and shaking the injected paw was recorded for a 5‐minute time period, as previously described.^31^ Nociception induced by safranal (20 nmol) was also evaluated 60 minutes after intraperitoneal (i.p.) treatment with HC‐030031 (100 mg/kg) or 30 minutes after capsazepine (4 mg/kg) or HC‐067047 (10 mg/kg) or their vehicle (all, 4% DMSO plus 4% tween 80 in isotonic saline, 0.9% NaCl). In another experimental setting, safranal (0.5‐1 mg/kg, i.g.) was administered each day for 5 consecutive days.^8^ Each day, 60 minutes after safranal i.g. administration, AITC (10 nmol), capsaicin (0.2 nmol) or GSK1016790A (2 nmol) or their vehicles (0.5% DMSO) were administered (20 μL, i.pl.) and acute nociceptive response was recorded.

### 2.8 Drugs and reagents

HC‐030031 [2‐(1,3‐dimethyl‐2,6‐dioxo‐1,2,3,6‐tetrahydro‐7H‐purin‐7‐yl)‐*N*‐(4‐isopropylphenyl) acetamide] was synthesized as previously described.^32^ If not otherwise indicated, all other reagents were obtained from Sigma‐Aldrich (Milan, Italy).

### 2.9 Statistical analysis

Statistical analysis was performed using the unpaired two‐tailed Student's *t* test for comparisons between two groups and the one‐ or two‐way ANOVA, followed by the post‐hoc Bonferroni's test for comparisons of multiple groups (GraphPad Prism version 5.00, San Diego, CA, USA). A *P* < 0.05 was considered statistically significant. Data are reported as mean ± SEM.

## 3 RESULTS

### 3.1 Safranal and picrocrocin, but not crocin, selectively activate the human TRPA1 channel

**Figure 1:**
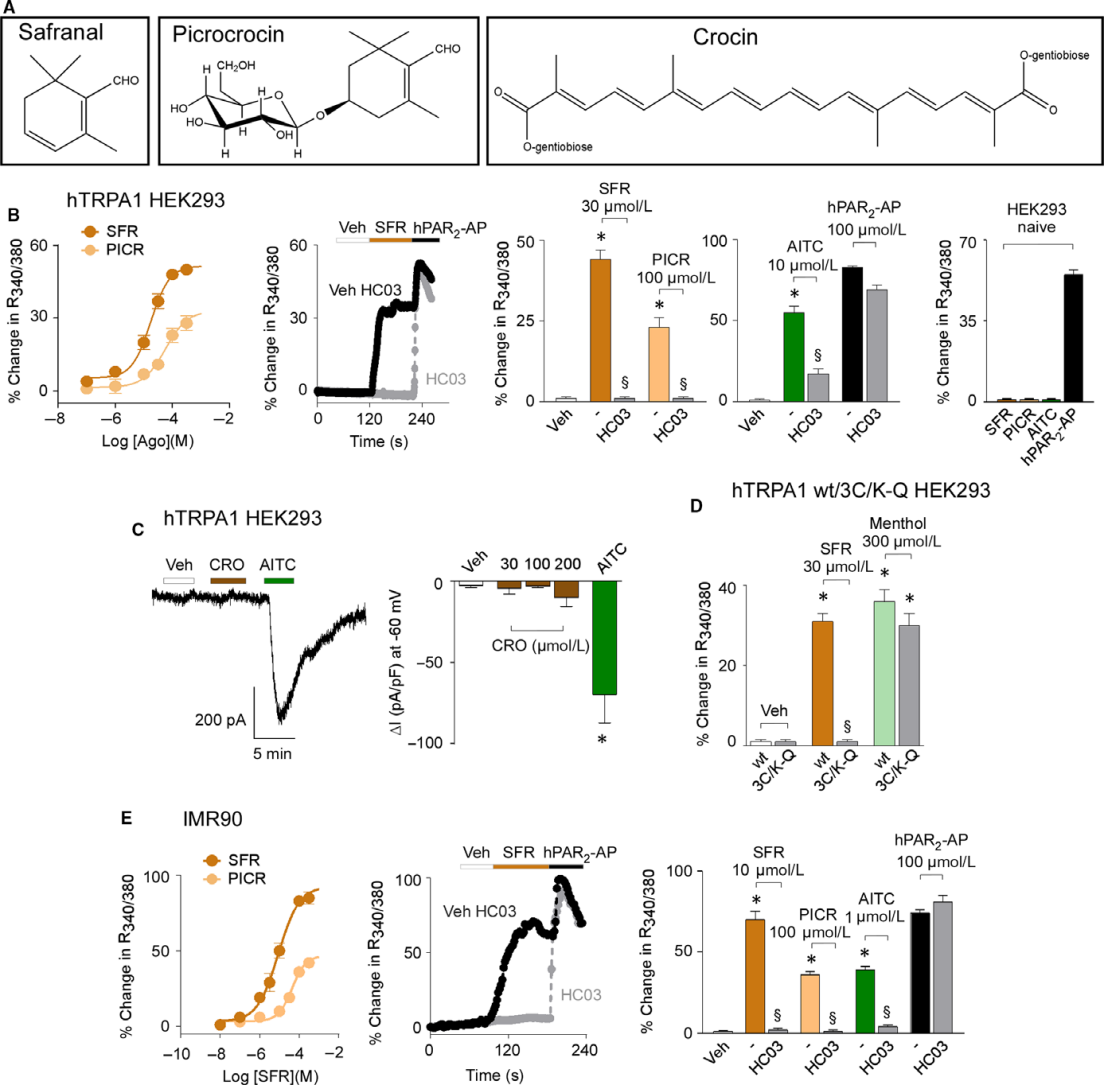
Safranal (SFR) and picrocrocin (PICR) selectively target the human TRPA1 channel. A, Chemical structures of SFR, PICR and crocin (CRO). B, Concentration response curves of the calcium mobilization evoked by SFR and PICR in hTRPA1 HEK293 cells. Representative traces and pooled data of calcium response evoked by SFR, PICR and AITC in hTRPA1 HEK293 pre‐exposed to HC‐030031 (HC03, 30 μmol/L) or its vehicle (‐) and in naïve HEK293 cells. C, Representative traces and pooled data of whole‐cell patch‐clamp inward currents evoked by CRO and AITC (100 μmol/L) in hTRPA1 HEK293. D, Pooled data of calcium responses evoked by SFR and menthol in wild‐type (wt) and mutant (3C/K‐Q) hTRPA1 HEK293 transfected cells. E, Concentration response curves of the calcium mobilization evoked by SFR and PICR in IMR90 cells. Representative traces and pooled data of calcium responses evoked by SFR, PICR and AITC pre‐exposed HC03 (30 μmol/L) or its vehicle (‐) in IMR90 cells. HC03 does not affect the response evoked by the hPAR_2_‐AP (100 μmol/L). Veh is the vehicle of SFR, dash (‐) indicates the vehicle of HC03. Data are mean ± SEM of n > 20 cells from 4 to 6 independent experiments (B, D, E) and n > 3 cells from 3 to 5 independent experiments (C). *P < 0.05 vs veh, ^§^ *P* < 0.05 vs SFR, PICR or AITC; one‐way ANOVA with Bonferroni post‐hoc correction

Safranal and picrocrocin evoked a concentration‐dependent calcium response in hTRPA1‐HEK293 (EC_50s_ 17 ± 0.3 μmol/L and 56 ± 0.3 μmol/L respectively), but not in naïve HEK293 cells (Figure 1B). The selective TRPA1 antagonist HC‐030031 attenuated the response evoked by safranal and picrocrocin and AITC (Figure 1B). Crocin, the third main constituent of saffron was not investigated in this test because of its intense yellow colour which interferes with the recording system. Thus, the ability of crocin to target TRPA1 was analysed by whole‐cell patch‐clamp electrophysiology. Crocin did not evoke any measurable inward current in hTRPA1‐HEK293, compared to its vehicle (Figure 1C).

Further investigation was limited to safranal, the most potent of the three compounds. The non‐electrophilic agonist, menthol, evoked a robust calcium response in 3C/K‐Q hTRPA1‐HEK293 cells, which express a mutant form of the TRPA1 receptor, lacking three key cysteine (C619, C639, C663) and one lysine (K708) residues, which are required for channel activation by electrophilic agonists^23^, ^33^ (Figure 1D). However, safranal failed to induce any calcium response in 3C/K‐Q hTRPA1‐HEK293 cells (Figure 1D). Finally, in IMR90 cells, which constitutively express the native human TRPA1 receptor,^34^ safranal and picrocrocin evoked concentration‐dependent calcium responses (EC_50s_ 9 ± 0.2 μmol/L and 44 ± 0.4 μmol/L respectively) that were attenuated by HC‐030031, alike the response evoked by AITC (Figure 1E).

Similar results were obtained in whole‐cell patch‐clamp recording experiments. Safranal elicited concentration‐dependent inward currents in hTRPA1‐HEK293 cells, an effect that was attenuated by HC‐030031 and was absent in untransfected HEK293 cells (Figure S1A). Either safranal or picrocrocin failed to evoke calcium responses or inward currents (safranal) in HEK293 cells transfected with the human TRPV1 (hTRPV1‐HEK293) or TRPV4 (hTRPV4‐HEK293) that, however, were efficiently stimulated by their selective agonists, capsaicin and GSK1016790A respectively (Figure S1B‐E). In all experiments, calcium responses elicited by hPAR2‐AP and ion currents evoked by KCl were not affected by HC‐030031, indicating selectivity (Figure 1B,E and Figure S1A).

### 3.2 Safranal selectively excites TRPA1 in rodent sensory neurons

**Figure 2:**
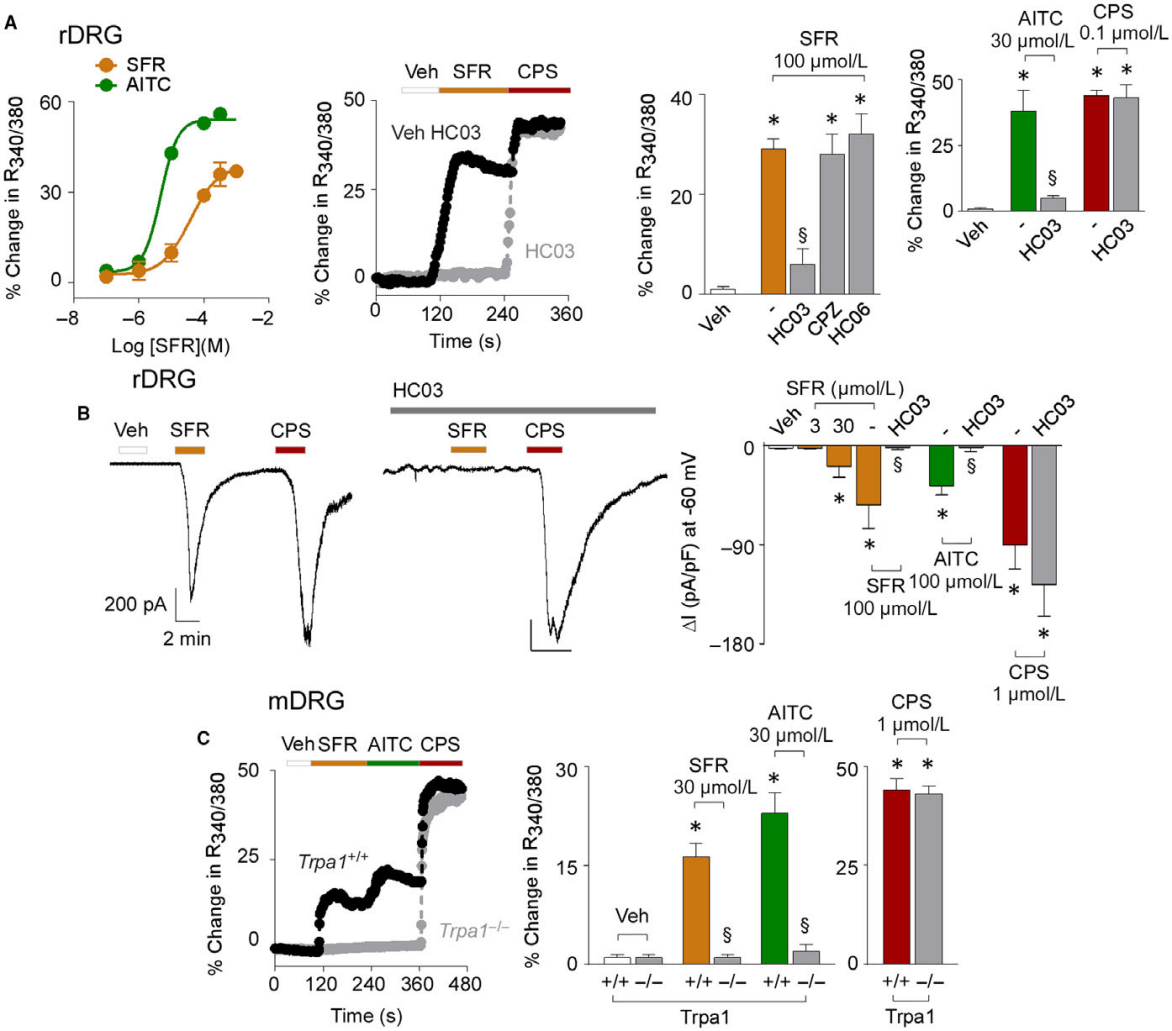
Safranal (SFR) selectively activates TRPA1 in rodent primary sensory neurons. A, Concentration response curves of the calcium mobilization evoked by SFR and AITC in rat DRG (rDRG) neurons. Representative traces and pooled data of calcium response evoked by SFR, AITC and capsaicin (CPS) in rDRG neurons pre‐exposed to HC‐030031 (HC03, 50 μmol/L), capsazepine (CPZ; 10 μmol/L), HC‐067047 (HC06; 30 μmol/L) or their vehicles (‐). B, Representative traces and pooled data of whole‐cell patch‐clamp inward currents evoked by SFR, AITC and CPS in rDRG neurons. HC03 does not affect the responses evoked by CPS. C, Representative traces and pooled data of the calcium responses evoked by SFR or AITC in mouse DRG (mDRG) neurons from *Trpa1* ^+/+^ and *Trpa1* ^−/−^ mice. Veh is the vehicle of SFR. Dash (‐) indicates vehicles of the different treatments. Data are mean ± SEM of n > 20 cells from 4 to 6 independent experiments (A, C) and n > 3 cells from 3 to 5 independent experiments (B). *P < 0.05 vs veh; ^§^ *P* < 0.05 vs SFR or AITC; one‐way ANOVA with Bonferroni post‐hoc correction

Exposure to safranal of cultured rat DRG neurons evoked a concentration‐dependent calcium response in a subset of cells, identified as nociceptors by their ability to respond to KCl, capsaicin and AITC^31^ (Figure 2A). In rat DRG neurons maximum calcium responses to safranal or AITC were 35.8% ± 8.5% and 56.0% ± 6.0% of ionomycin (n = 25, *P* < 0.01) respectively, and EC_50s_ were 38 ± 0.03 μmol/L and 5 ± 0.3 μmol/L respectively. Calcium responses were attenuated by HC‐030031, and unaffected by the respective TRPV1 and TRPV4 selective antagonists, capsazepine and HC‐067047 (Figure 2A). Safranal or AITC elicited inward currents in rat DRG neurons, that were blocked by HC‐030031 (Figure 2B), which, however, did not affect currents evoked by capsaicin, indicating selectivity (Figure 2A,B). Safranal and AITC produced calcium responses in DRG neurons isolated from *Trpa1* ^+/+^ mice, but not in neurons isolated from *Trpa1* ^−/−^ mice (Figure 2C), while the response to capsaicin was unchanged in both mice strains.

### 3.3 Safranal causes neuropeptides release and acute pain via TRPA1 activation in nociceptors

There is evidence that TRPA1 is localized in peptidergic primary sensory neurons.^35^, ^36^ Central and peripheral endings of primary sensory neurons are widely expressed in most tissues and organs, including rat urinary bladder and rat or mouse spinal cord, where upon stimulation they release proinflammatory neuropeptides, such as CGRP and SP. SP released upon stimulation of TRPV1 or TRPA1 results in a contractile response of isolated strips of rat urinary bladder that is mediated by activation of the NK1 and NK2 receptors for SP in bladder smooth muscle cells.^37^

**Figure 3:**
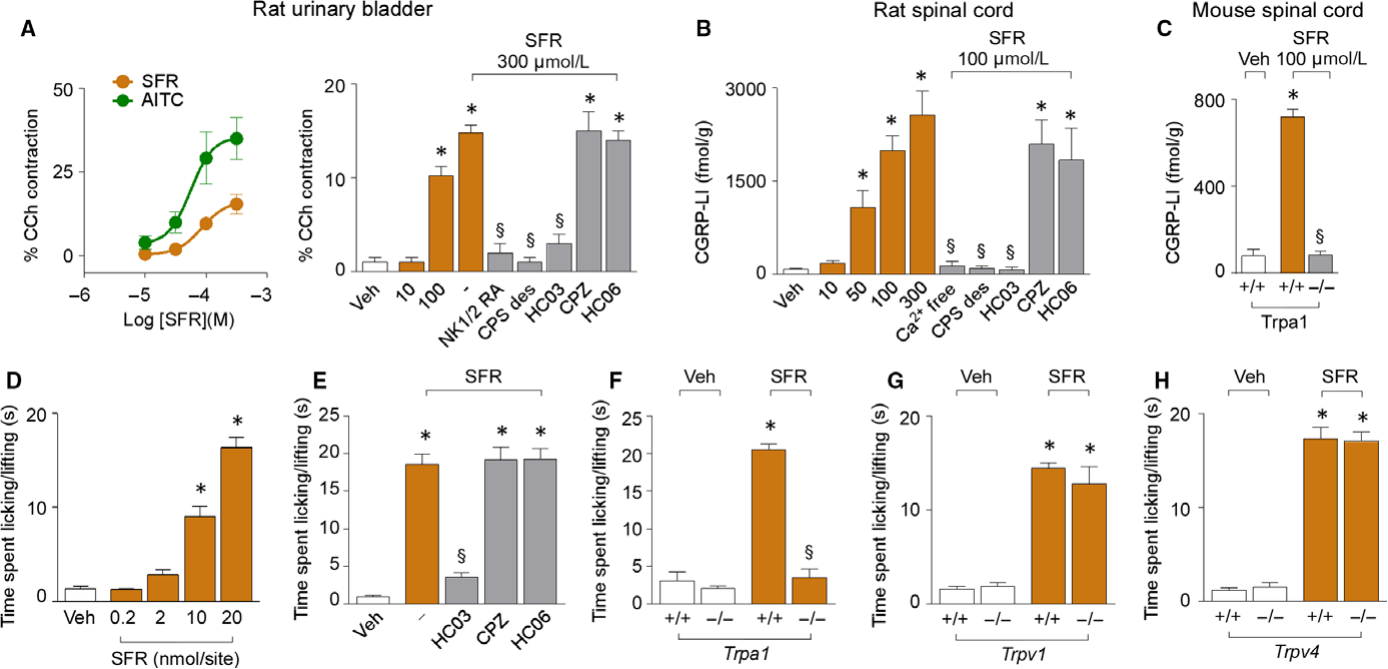
Safranal (SFR) activating TRPA1 in nociceptors causes neuropeptides release and pain. A, Concentration response curves of the contractile response induced by SFR and AITC in rat urinary bladder. Pooled data of the contractile response evoked by SFR in rat urinary bladder pre‐exposed to a combination of NK1/NK2 receptor antagonists (NK1/2 RA; L‐733,060 and SR48968, both 1 μmol/L), to a high concentration of capsaicin (CPS des; 10 μmol/L), HC‐030031 (HC03, 50 μmol/L), capsazepine (CPZ; 10 μmol/L), HC‐067047 (HC06; 30 μmol/L) or their vehicles (‐). B, Pooled data of CGRP release evoked by SFR from rat dorsal spinal cord after calcium removal (Ca^2+^ free), or after capsaicin desensitization (CPS des; 10 μmol/L), or exposed to HC03 (50 μmol/L), CPZ (10 μmol/L) or HC06 (30 μmol/L). (C) CGRP release evoked by SFR from dorsal spinal cord from *Trpa1* ^+/+^ and *Trpa1* ^−/−^ mice. D, Dose‐dependent acute nociceptive response evoked by intraplantar (i.pl.) injection (20 μL) of SFR in C57BL/6 mice. E, Pooled data of the acute nociceptive response evoked by SFR (20 nmol, i.pl.) after intraperitoneal (i.p.) HC03 (100 mg/kg), CPZ (4 mg/kg), HC06 (10 mg/kg) or their vehicles (‐, 4% DMSO, 4% tween 80 in 0.9% NaCl). F‐H, Acute nociceptive response evoked by SFR (20 nmol, i.pl.) in *Trpa1* ^+/+^ and *Trpa1* ^−/−^ *, Trpv1* ^+/+^ and *Trpv1* ^−/−^, and *Trpv4* ^+/+^ and *Trpv4* ^−/−^ mice. Veh is the vehicle of SFR. Dash (‐) indicates vehicles of treatments. Data are mean ± SEM of 4‐6 independent experiments (A‐C) and n = 6 mice per group (D‐H). *P < 0.05 vs Veh; ^§^ *P* *<* 0.05 vs SFR; one‐way ANOVA with Bonferroni post‐hoc correction

Safranal caused a concentration‐dependent contractile response of isolated strips of rat urinary bladder with a slightly lower potency than AITC (EC_50s_ were 76 ± 0.07 μmol/L and 56 ± 0.02 μmol/L respectively) (Figure 3A). Efficacy of safranal was also lower than that of AITC (maximum response, 15.5% ± 3.0% of carbachol, n = 6, and 35.1% ± 6.2% of carbachol respectively, n = 6, *P* < 0.05) (Figure 3A). The response of safranal was attenuated by pre‐exposure to a combination of NK1 and NK2 receptor antagonists (L‐733,060 and SR48968 respectively), by pre‐exposure to a high concentration of capsaicin able to desensitize the nociceptors and by pre‐exposure to the TRPA1 selective antagonist, HC‐030031, while it was unaffected by pre‐exposure to capsazepine (TRPV1 antagonist) and HC‐067047 (TRPV4 antagonist) (Figure 3A). This finding supports the hypothesis that safranal behaves as a partial agonist at the TRPA1 channel.

Safranal elicited a concentration‐dependent increase in CGRP‐LI outflow from rat spinal cord slices which was absent in a calcium‐free medium or in tissues pre‐exposed to a desensitizing concentration of capsaicin and in the presence of HC‐030031 (Figure 3B). Safranal‐evoked CGRP release was unaffected by capsazepine or HC‐067047 (Figure 3B). Exposure to safranal increased CGRP outflow from dorsal spinal cord slices obtained from *Trpa1* ^+/+^ mice, but not from tissues taken from *Trpa1* ^−/−^ mice (Figure 3C). Thus, safranal elicits CGRP release from a subset of TRPV1‐positive neurons via a neurosecretory process, mediated by TRPA1.

These ex vivo findings were replicated in an in vivo setting. Injection of safranal (0.2‐20 nmol) into the mouse paw (20 μL, i.pl.) evoked a dose‐dependent acute nociceptive response (Figure 3D). The response evoked by the highest dose (20 nmol) of safranal was attenuated by pretreatment with systemic administration HC‐030031 (100 mg/kg, i.p.) and was absent in *Trpa1* ^−/−^ mice (Figure 3E,F). Selectivity of safranal action for TRPA1 was strengthened by the observation that the nociceptive response evoked by i.pl. safranal (20 nmol) in *Trpv1* ^+/+^ and *Trpv4* ^+/+^ was maintained in *Trpv1* ^−/−^ and *Trpv4* ^−/−^ mice (Figure 3G,H). Similarly, the nociceptive response to safranal was unaffected by pretreatment with TRPV1 and TRPV4 antagonists (capsazepine and HC‐067047 respectively) (Figure 3E).

### 3.4 In vitro and in vivo exposure to safranal attenuated TRPA1‐mediated responses

**Figure 4:**
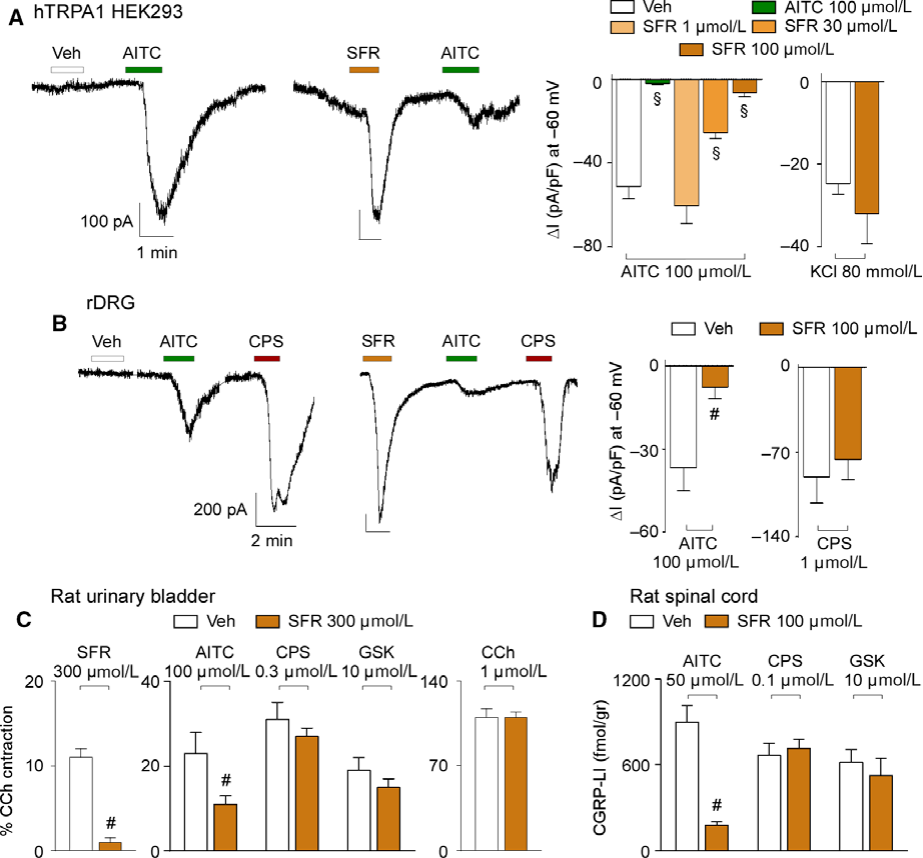
Safranal (SFR) causes desensitization. A, Representative traces and pooled data of whole‐cell patch‐clamp inward currents of the concentration dependant desensitization induced by SFR in response to AITC and KCl in hTRPA1 HEK293. B, Representative traces and pooled data of whole‐cell patch‐clamp inward currents of the desensitization induced by SFR in response to AITC and capsaicin (CPS) in rat DRG (rDRG) neurons. C, Pooled data of the desensitization induced by SFR in the contractile response evoked by SFR, AITC, CPS, GSK and carbachol (CCh). D, Pooled data of the desensitization induced by SFR in CGRP‐LI release from rat spinal cord evoked by AITC, CPS and GSK. Veh is the vehicle of SFR. Data are mean ± SEM of n > 3 cells from 3‐5 independent experiments (A, B) and 4‐6 independent experiments (C, D). ^§^ *P* < 0.05 vs AITC, SFR; one‐way ANOVA followed by Bonferroni test. ^#^ *P* < 0.05 vs AITC and SFR, Student's *t* test

In hTRPA1‐HEK293 transfected cells, inward currents evoked by AITC underwent a concentration‐dependent attenuation after pre‐exposure to increasing concentrations of safranal (Figure 4A), whereas KCl‐evoked currents were not affected (Figure 4A). In cultured rat DRG neurons, pre‐exposure to safranal reduced inward currents evoked by AITC, but not currents evoked by capsaicin, suggesting that safranal promotes selective TRPA1 desensitization (Figure 4B). Specific desensitization of TRPA1 was also observed in organ bath experiments. Pre‐exposure to safranal reduced contractile responses of rat urinary bladder strips evoked by safranal and AITC, but not those evoked by capsaicin, GSK1016790A or carbachol (Figure 4C). Finally, pre‐exposure to safranal was able to markedly reduce CGRP release from rat spinal cord evoked by AITC, without affecting the release evoked by capsaicin or GSK1016790A (Figure 4D).

**Figure 5:**
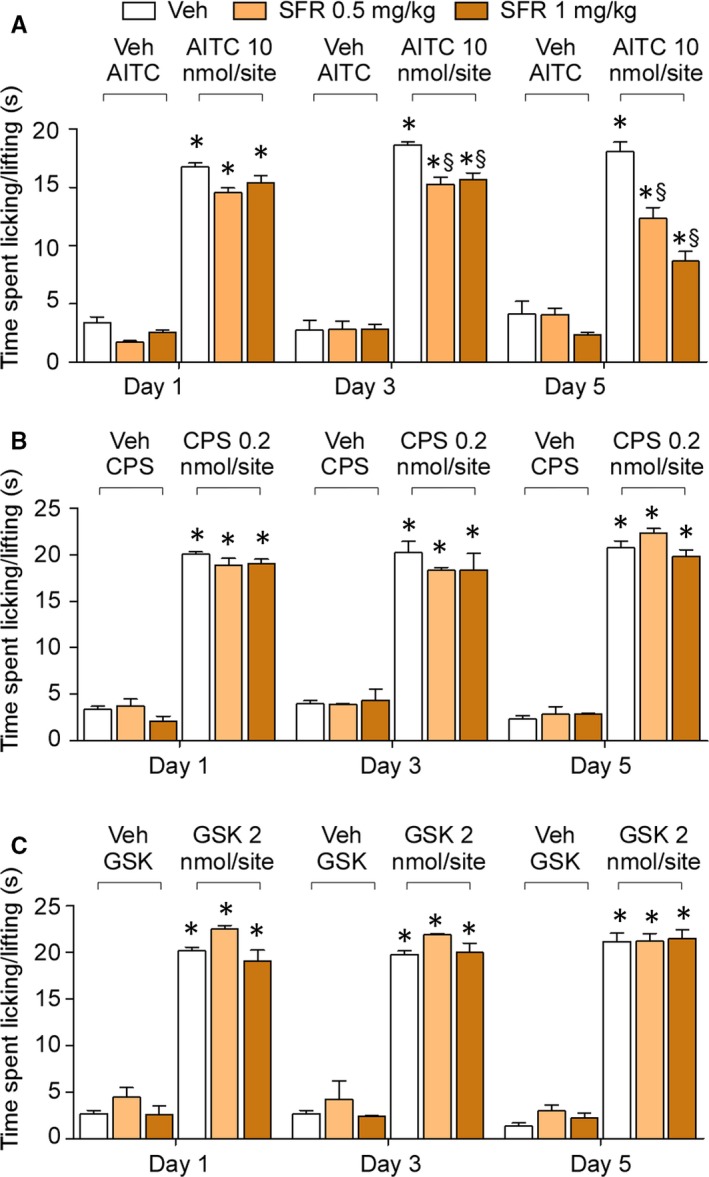
Repeated treatment with systemic safranal (SFR) causes TRPA1 desensitization. A‐C, Pooled data of the acute nociceptive response induced by intraplantar (20 μL) AITC (A), capsaicin (CPS) (B) or GSK1016790A (GSK) (C) after daily intragastric administration of SFR (0.5‐1 mg/kg) in C57BL/6 mice. Veh is vehicle of SFR. Data are mean ± SEM of n = 6 mice per group. *P < 0.05 vs veh; ^§^ *P* < 0.05 vs AITC; two‐way ANOVA with Bonferroni post‐hoc correction

To test whether pre‐exposure to safranal could desensitize TRPA1‐mediated responses in vivo, safranal was administered by the intragastric route (i.g.) at two different doses (0.5‐1 mg/kg). One single i.g. administration of either doses (day 1) did not affect the ability of local (i.pl.) AITC, capsaicin or GSK1016790A to evoke acute nociceptive responses (Figure 5A‐C). After administration of the two doses of safranal for 3 consecutive days the nociceptive responses evoked by i.pl. AITC were slightly reduced, but not those evoked by capsaicin and GSK1016790A (Figure 5A‐C). After administration of the two doses of safranal for 5 consecutive days the nociceptive responses evoked by i.pl. AITC were markedly attenuated, the effect of the highest dose of safranal being more pronounced (Figure 5A). Nociceptive responses evoked by both capsaicin and GSK1016790A were unaffected (Figure 5B,C).

## 4 DISCUSSION

Present results show that two of the three main constituents of saffron, safranal and its precursor, picrocrocin, target the mouse, rat and human (native and recombinant) TRPA1, whereas the third constituent, crocin, was completely inactive. The lower potency of picrocrocin compared to safranal may depend on various factors. The presence of two β substituents on the double bond and chemical instability as compared to safranal^38^ could explain the lower reactivity of picrocrocin. The failure to stimulate TRPV1 or TRPV4 channels indicates that nociceptor activation by safranal is selectively mediated by TRPA1. Interestingly, safranal failed to evoke any calcium response in HEK293 cells transfected with mutant TRPA1 (3C/K‐Q) channel, indicating that, similarly to other electrophilic and reactive agonists,^23^, ^33^ channel activation by the saffron constituent is mediated by specific cysteine/lysine residues. Both potency and efficacy of the calcium response evoked by safranal in cultured rat DRG neurons were lower than that of AITC. In particular, the lower efficacy supports the hypothesis that safranal may act as a partial TRPA1 agonist.

Peptidergic sensory neurons expressing TRPA1 exert a dual afferent and efferent role, because upon stimulation they can both signal pain to the brain and release from their peripheral terminals the neuropeptides SP and CGRP, which mediate neurogenic inflammatory responses.^13^, ^39^ One of these responses is the SP‐mediated contraction of rat urinary bladder smooth muscle,^40^ which can be also produced by TRPA1 activation in intramural sensory nerve terminals.^37^ Here, we found that safranal produced a contraction mechanistically similar to that induced by AITC, because it was abrogated after exposure to a high, desensitizing, concentration of capsaicin, in the presence of a TRPA1 selective antagonist and in the presence of a combination of NK1 and NK2 receptor antagonists. Notably, the efficacy of safranal to target TRPA1 in the urinary bladder was lower than that of AITC, further supporting a possible partial agonism of the compound.

Results obtained in vitro were recapitulated in an in vivo setting, where the proalgesic action of safranal was mediated exclusively by TRPA1. Intraplantar injection of safranal elicited a concentration‐dependent acute nociceptive response that was TRPA1‐dependent, being selectively attenuated by TRPA1 antagonism and gene deletion. However, there is also indication that saffron has beneficial effect in certain pain conditions.^3^, ^41^ Furthermore, safranal has been reported to attenuate pain‐like responses in animal models of inflammatory and neuropathic pain.^8^, ^9^, ^10^ In particular, safranal attenuated mechanical allodynia and thermal hyperalgesia in a chronic constriction injury model,^10^ suppressed the second phase of the orofacial pain induced by formalin^42^ and the late phase of the carrageenan‐induced paw oedema.^8^ The apparent contradiction between these findings and the ability of safranal to excite the proalgesic TRPA1 may be explained considering that pre‐exposure to safranal of cultured DRG neurons selectively attenuated AITC‐evoked responses, without affecting TRPA1‐independent responses. In vitro results were recapitulated by in vivo experiments, where repeated administration (5 days) of i.g. safranal reduced the acute nociceptive responses evoked by local AITC, leaving unaffected the nociceptive responses evoked by TRPV1 or TRPV4 stimulation. Thus, safranal attenuates the afferent function of nociceptors apparently by promoting a process of homologous desensitization of the TRPA1 channel, which might contribute to the antinociceptive properties of saffron. More recently, it has been demonstrated that TRPA1 is expressed by Schwann cells, where it can amplify and sustain macrophage‐dependent neuropathic pain.^19^ Thus, it is possible that safranal exerts its partial agonist and desensitizing activities at the Schwann cell TRPA1, contributing in this manner to reduce neuropathic pain.

Clinical investigations with saffron has reported headache as a possible adverse reaction.^4^ In contrast with this observation, saffron has been used by Indian traditional medicine to treat headaches.^5^ TRPA1 is preferentially expressed by peptidergic sensory neurons, and upon its activation evokes the simultaneous release of the proinflammatory and proalgesic neuropeptides, SP and CGRP.^21^, ^43^ CGRP is now considered a major contributor of migraine pain as small molecule CGRP receptor antagonists and monoclonal antibodies against CGRP or its receptor have marked beneficial effects in migraine.^13^, ^15^ The observation that in strips rat urinary bladder and in rat dorsal spinal cord slices pre‐exposure to safranal reduced AITC‐evoked SP‐mediated contractile responses and CGRP release respectively, without affecting TRPA1‐independent responses further suggests that safranal attenuates the efferent and pro‐migraine function of peptidergic nociceptors.

We previously showed that upon in vitro or in vivo exposure to other herbal preparations, such as isopetasin, contained in butterbur [*Petasites hybridus* (L.) Gaertn.],^29^ and parthenolide, a major constituent of *Tanacetum parthenium*,^30^ TRPA1‐expressing trigeminal neurons undergo concentration‐ or dose‐dependent desensitization. However, while isopetasin and parthenolide evoked non‐selective desensitization of peptidergic nociceptors as they also attenuated responses mediated by TRPV1 and TRPV4 activation,^29^, ^30^ safranal seems to selectively reduce responses elicited by TRPA1 agonism. The mechanism of the selective activity of safranal is unknown. Different pharmacokinetic properties or distinct activation of intracellular signalling mechanisms may be the causes of the diverse ability of the three herbal derivatives to desensitize the channel, and these deserve further investigation. Nevertheless, safranal, one major constituent of saffron extract, targets TRPA1 with a lower potency than full agonists and attenuates responses mediated by TRPA1 activation by other stimuli. The dual action of safranal on TRPA1 might contribute to the reported either detrimental or beneficial actions of the compound in animal models of pain and of saffron in humans.

## CONFLICT OF INTEREST

RP is fully employed at Chiesi Farmaceutici SpA, Parma, Italy. The other authors declare no competing financial interests.

## AUTHOR CONTRIBUTION

SLP, LL, EC, RP, PG, SM, RN and FDL designed experiments and interpreted results. SLP, LL, SJM and VS, performed calcium experiments. SLP, EC and R.P., performed electrophysiological experiments. SLP, LL, SJM, IMM, EC and SM, performed neurochemical in vitro assays. SLP, LL, SJM, IMM, SM, RN, and FDL, performed in vivo experiments. RP, PG, RN and FDL wrote the manuscript.
