# Supplementary material for: TRPA1 mediates the antinociceptive properties of the constituent of Crocus sativus L., safranal
Source: J Cell Mol Med. 2019 Jan 12;23(3):1976–86. doi: 10.1111/jcmm.14099 (PMC6378183; doi:10.1111/jcmm.14099)
Supplement: Supplementary file 1 [file JCMM-23-1976-s001.docx]

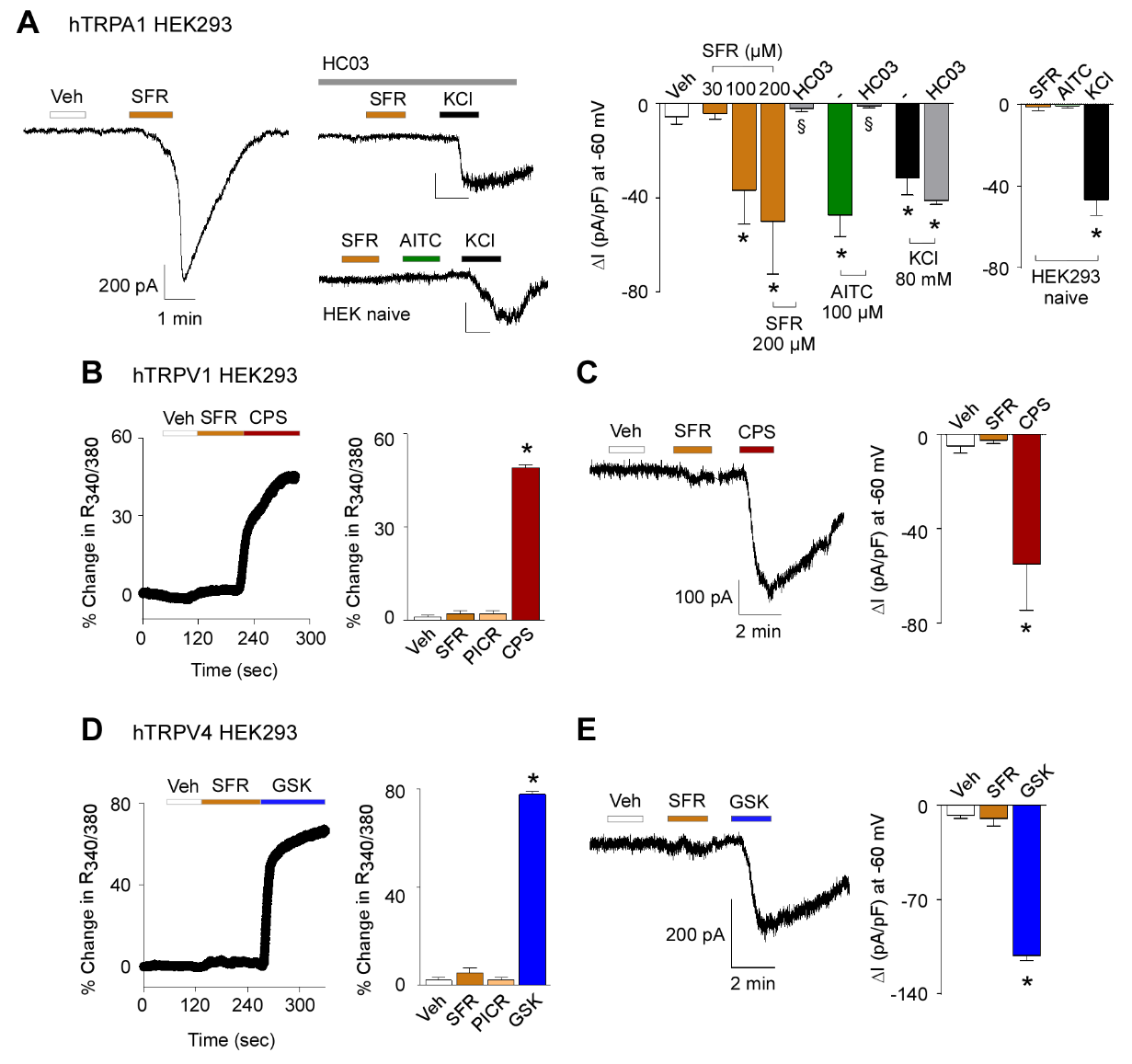


**Figure S1.** (A) Representative traces and pooled data of whole-cell patch-clamp inward currents evoked by SFR, AITC and KCl in hTRPA1 HEK293 cells pre-exposed to HC-030031 (HC03, 30 µM) or its vehicle (-) and in naïve HEK293 cells. (B, C) Representative traces and pooled data of calcium responses and whole-cell patch-clamp inward currents evoked by SFR (100 µM), PICR (200 µM) and capsaicin (CPS; 1 µM) in hTRPV1 HEK293. (D, E) Representative traces and pooled data of calcium responses and whole-cell patch-clamp inward currents evoked by SFR (100 µM), PICR (200 µM) and GSK1016790A (GSK; 0.5-1 µM) in hTRPV4 HEK293. Veh is vehicle of SFR. Data are mean ± SEM of n > 4 cells from 4-6 independent experiments (A, C, E) and n > 30 cells from 4-6 independent experiments (B, D). **P* < 0.05 *vs*. veh; ^§^*P* < 0.05 *vs*. SFR and AITC; one-way ANOVA followed by Bonferroni test.
