# Supplementary figures and images for: TRPA1 mediates the antinociceptive properties of the constituent of Crocus sativus L., safranal
Source: J Cell Mol Med. 2019 Jan 12;23(3):1976–86. doi: 10.1111/jcmm.14099 (PMC6378183; doi:10.1111/jcmm.14099)

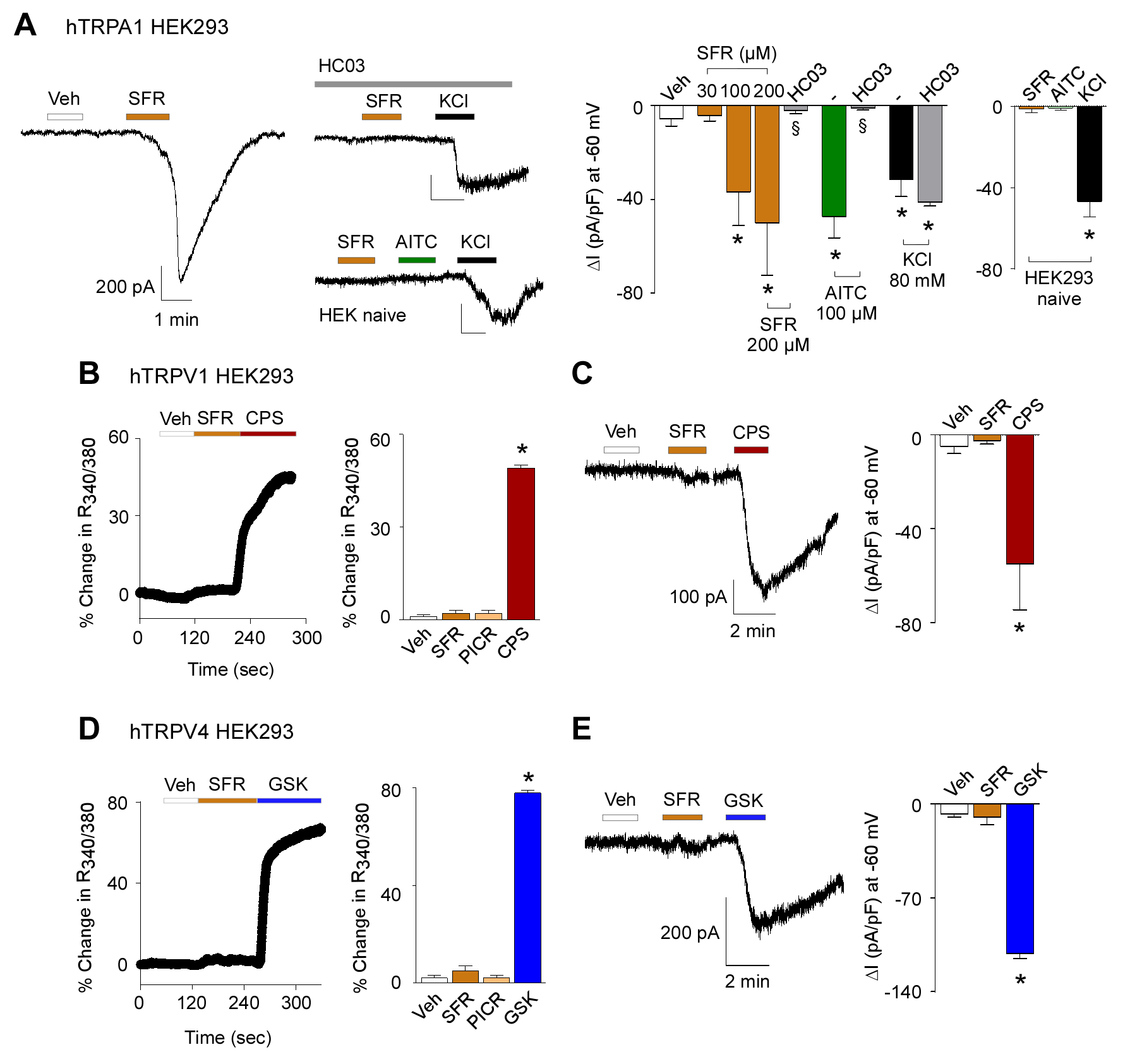

Supplement: Supplementary file 2 [file JCMM-23-1976-s002.tif]
